# Two paths to the body’s superhighway—the auricular branch (taVNS) and the cervical bundle (tcVNS): a narrative review

**DOI:** 10.3389/fnins.2026.1848801

**Published:** 2026-07-15

**Authors:** Peter S. Staats, Justyna Blake

**Affiliations:** 1 Vagus Nerve Society, Atlantic Beach, FL, United States; 2 electroCore, Inc. Rockaway, NJ, United States

**Keywords:** auricular vagus nerve stimulation, cervical vagus nerve stimulation, vagus, vagus afferent nerves, vagus nerve (VN) stimulation

## Abstract

Interest in the vagus nerve has recently surged, particularly with the advent of non-invasive transcutaneous VNS (tVNS), which has broadened access and facilitated research across a range of conditions including depression, post-traumatic stress disorder (PTSD), migraine, chronic pain, and inflammatory disorders. tVNS primarily utilizes two pathways: the auricular branch (taVNS) and the cervical bundle (tcVNS). This paper provides a narrative review of tcVNS and taVNS stimulation. While both target the same cranial nerve, they differ significantly in their anatomical access points and methodological implementations.

## Introduction

Long before the advent of modern neuroscience and the formal discovery of the vagus nerve’s role in regulating the body’s internal state, ancient healing modalities such as Traditional Chinese Medicine and Ayurveda systematically targeted pathways now recognized as vagal networks. Ayurvedic Pranayama (breath regulation) relies on extended exhalations to slow down heart rate, mechanically triggering parasympathetic activation and improving heart rate variability (HRV). Concurrently, traditional acupuncture targets anatomical zones packed with sensory nerve fibers. Specifically, stimulating the cavum conchae of the ear engages the auricular branch of the vagus nerve (ABVN), driving signals directly to the brainstem to downregulate systemic stress and inflammation. These methods lack the modern anatomical term “vagus nerve,” but their core principles and practices successfully modulate the body’s internal state in ways that modern science attributes to vagus nerve stimulation (Lv et al., 2022).

In the late 19th century, inspired by electrotherapy, James Corning proposed the concept of vagus nerve stimulation (VNS) and attempted to treat epilepsy by modulating neuronal excitability through electrical stimulation of the vagus nerve (Lanska, 2002). Although early efforts did not achieve clinical success, subsequent animal studies by Zabara revealed the potential of VNS demonstrating the ability to interrupt seizures and prolong seizure suppression in canine epilepsy models (Zabara, 1992). In 1988, the world’s first VNS surgery was performed, and this foundational work ultimately led to regulatory approval. In 1997, the Food and Drug Administration (FDA) approved the first implantable VNS device (iVNS) for the treatment of drug-resistant epilepsy (Penry and Dean, 1990). Since then, iVNS has expanded, with approvals now including epilepsy, treatment-resistant depression, stroke rehabilitation, and rheumatoid arthritis (Nemeroff et al., 2006; Schambra and Hays, 2025; Uthman et al., 2004).

While VNS is an established treatment, access to VNS has been limited over the first few decades of use due to the invasive and expensive nature of iVNS (Austelle et al., 2024; Lv et al., 2022). Consequently, non-invasive transcutaneous VNS (tVNS) has been developed as a less expensive, safe and easily applicable alternative. This has opened the door for scientists to study the effects of vagal stimulation more closely and has moved this area forward, making the therapy more accessible and driving widespread interest across both clinical and wellness communities (Austelle et al., 2024). As understanding of the vagus nerve deepens, it has increasingly become a focal point in the search for holistic, nervous system-based treatments that bridge ancient practices and cutting-edge biomedical technology.

## Electrical non-invasive vagus nerve stimulation

Transcutaneous auricular vagus nerve stimulation (taVNS) and transcutaneous cervical vagus nerve stimulation (tcVNS) are two forms of non-invasive electrical vagus nerve stimulation. Both taVNS and tcVNS aim to activate the afferent fibers of the vagus nerve, sending signals to the brainstem to modulate activity in other brain areas. However, they differ significantly in their anatomical access points and methodological implementations.

### Auricular vagus nerve stimulation

The ABVN—also known as Arnold’s nerve—is the only peripheral branch of the vagus nerve and follows a distinctive anatomical course to reach the external ear and supply cutaneous sensation. It originates from the superior (jugular) ganglion of the vagus nerve as it exits the jugular foramen at the base of the skull. After branching off, it briefly communicates with the facial nerve (CN VII) and enters the mastoid canaliculus, a small canal in the lateral wall of the jugular fossa. From there, it re-enters the temporal bone and then exits the skull through the tympanomastoid fissure, located between the mastoid process and the tympanic part of the temporal bone, just behind the ear (Peuker and Filler, 2002).

**FIGURE 1:**
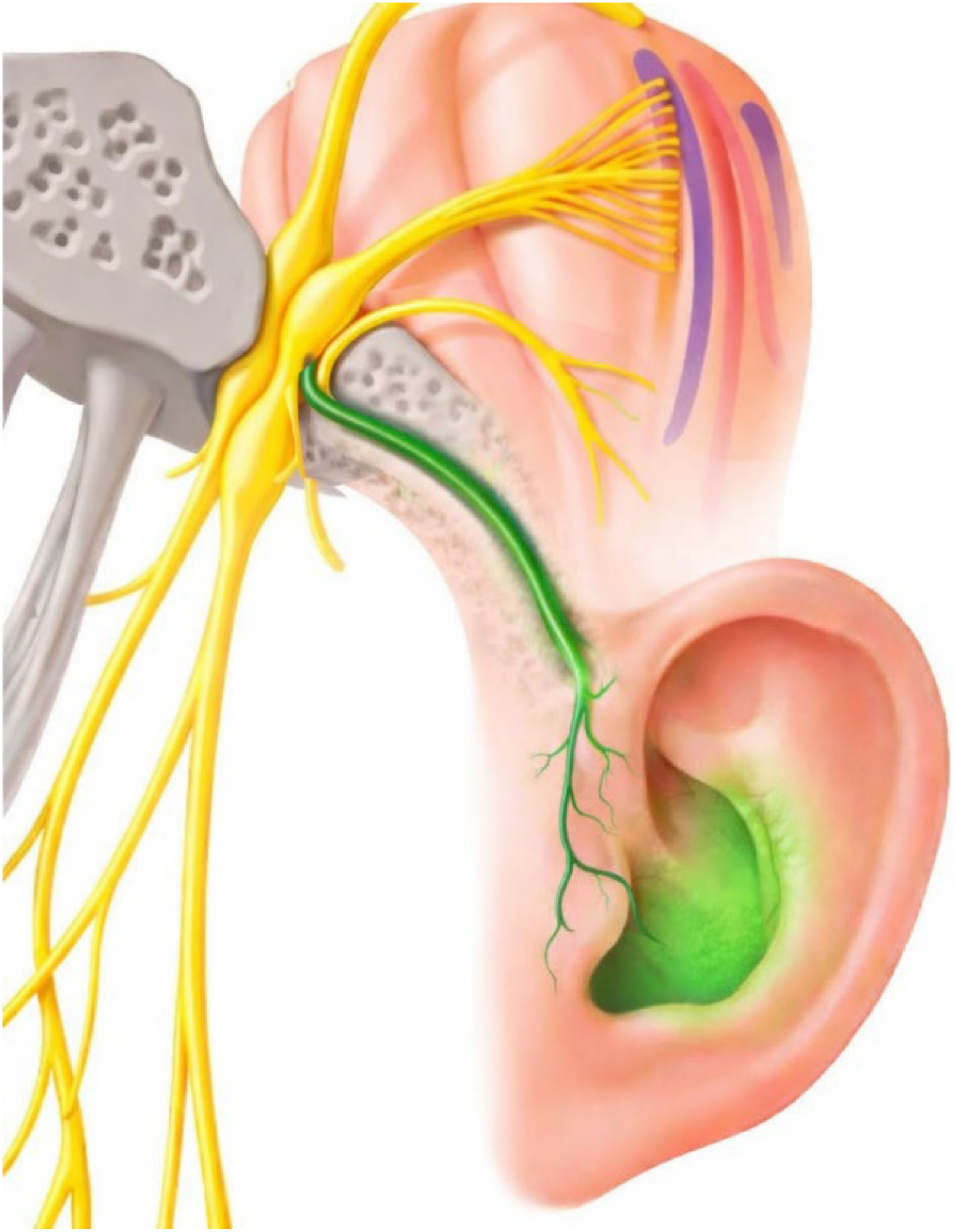
Cutaneous distribution of auricular vagus nerve innervation in the external ear. Illustration of the regions of the external ear innervated by the auricular branch of the vagus nerve, including the concha, cymba conchae, posterior wall of the external auditory canal, and portions of the tympanic membrane. These areas represent the primary targets for electrode placement in taVNS. The Figure also highlights the overlap with non-vagal sensory innervation (e.g., auriculotemporal and greater auricular nerves), emphasizing the anatomical variability and challenges associated with selective vagal stimulation at the auricle. Image used under license from Kenhub GmbH. Modified by Licensee.

Upon exiting, the auricular branch innervates specific regions of the outer ear, including the concha, cymba conchae, posterior wall of the external auditory canal, and occasionally the external surface of the tympanic membrane. Importantly auricular vagal fibers are entirely afferent, and efferent stimulation must come from a reflex activation at the trigeminal islands, abutting the vagal complex (Austelle et al., 2024; Bermejo et al., 2017; Butt et al., 2020; Kaniusas et al., 2019; Kenny and Bordoni, 2025; Lv et al., 2022; Peuker and Filler, 2002; Tekdemir et al., 1998). See Figure 1.

Transcutaneous auricular vagus nerve stimulation is administered through electrodes placed on specific areas of the outer ear, typically the tragus or the cymba conchae, areas of the ear innervated by the ABVN. The earlobe is often used for sham stimulation, as it has minimal ABVN innervation (Kenny and Bordoni, 2025). Stimulation may be applied to the left, right, or both ears. Research has traditionally favored the left ear, primarily due to conventions established during decades of using invasive VNS devices, while some do explore the different effects of right-sided or bilateral stimulation. Selection of the stimulation site appears to be arbitrary, either predetermined by the device employed in the experiment or based on other previous studies (Yap et al., 2020a).

A stimulation unit, which can be part of a device that resembles an earbud, is used to deliver the electrical current. The device is typically clipped or fitted to specific areas of the ear. The electrodes applied to the surface of the skin are usually titanium since it is a durable and hypoallergenic material, making it a suitable choice for electrodes that have direct and prolonged contact with the skin. These electrodes deliver the electrical current to the vagus nerve endings in the ear. Many devices can be customized through a companion mobile app, allowing the user to adjust the intensity and frequency of the electrical pulses. Electrode design, configuration (monopolar/bipolar), target location and stimulation parameters vary widely, but most commonly used TaVNS waveform parameters are highly similar to the typical parameters used for the stimulation of the cervical vagus, at a pulsewidth of 250 us and frequency of 20 Hz. Many researchers agree that optimal parameters for taVNS are still being explored. Side-effects of taVNS are minimal, with skin irritation or redness being the most common side-effect (Kim et al., 2022).

**FIGURE 2:**
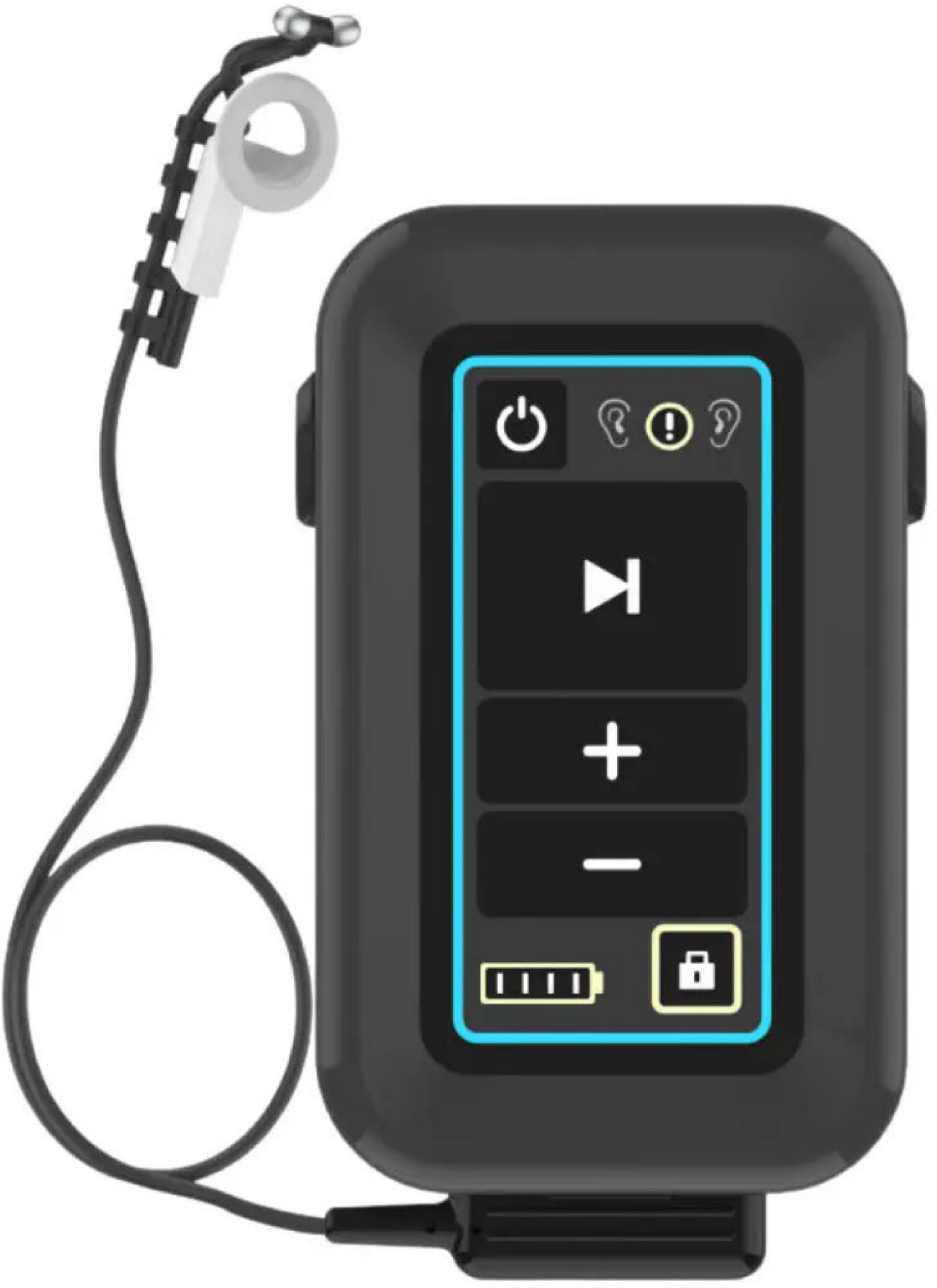
The NEMOS^®^ transcutaneous auricular vagus nerve stimulation (taVNS) system. The device includes a handheld stimulator unit and a dedicated ear electrode designed to target the auricular branch of the vagus nerve (ABVN) within the concha.

NEMOS^®^ (now marketed as the tVNS^®^ L by tVNS Technologies GmbH, previously Cerbomed), see Figure 2, is widely considered the first taVNS device to receive medical approval and remains one of the most established devices in clinical research (Farmer et al., 2021). The device targets the concha of the outer ear and is CE-marked (European Conformity) for the European market for use in the treatment of epilepsy, depression, chronic pain and anxiety (Yap et al., 2020b). This device does not yet have FDA clearance for these indications (tVNS Technologies GmbH, 2025).

It’s a handheld, battery-driven device connected to a specialized ear electrode made of titanium/titanium-iridium. Stimulation intensity can be adjusted by the patient, caregiver or treating physician (increased at steps of 0.1 mA until the perception threshold of the electrical stimulation is reached). Impedance is measured automatically and insufficient electrode contact with the skin evokes an alarm. During stimulation, a series of electrical pulses (pulse width: 250 μs, frequency: 25 Hz, duty cycle: 30 s on, 30 s off, to avoid habituation) are applied to the skin of the concha. Patients are asked to stimulate for a total of 4 h per day (Straube et al., 2015).

### Cervical vagus nerve stimulation

**FIGURE 3:**
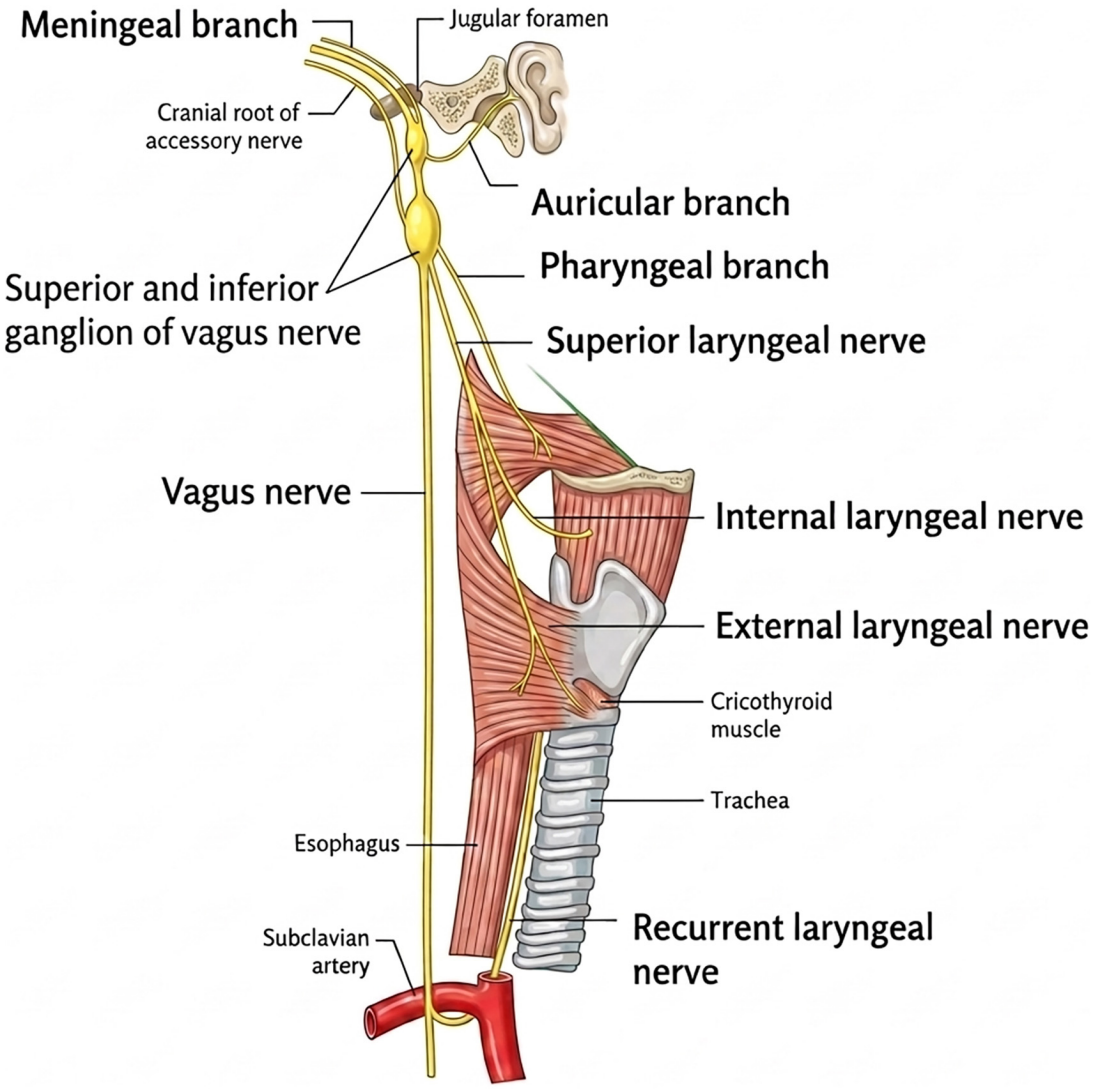
Anatomical course and major branches of the vagus nerve (CN X). The diagram illustrates the superior and inferior ganglia near the jugular foramen and the branching patterns in the cervical region, including the auricular branch.

After exiting the skull through the jugular foramen, the vagus nerve descends through the neck within the carotid sheath, positioned between the internal/common carotid artery and the internal jugular vein (see Figure 3). Here, it runs very close to the surface of the neck and can be reliably identified by palpation of the carotid artery. The vagus nerve can be stimulated by placing electrodes over the skin on the side of the neck, between the laryngeal prominence and the sternocleidomastoid muscle (Goadsby et al., 2014).

**FIGURE 4:**
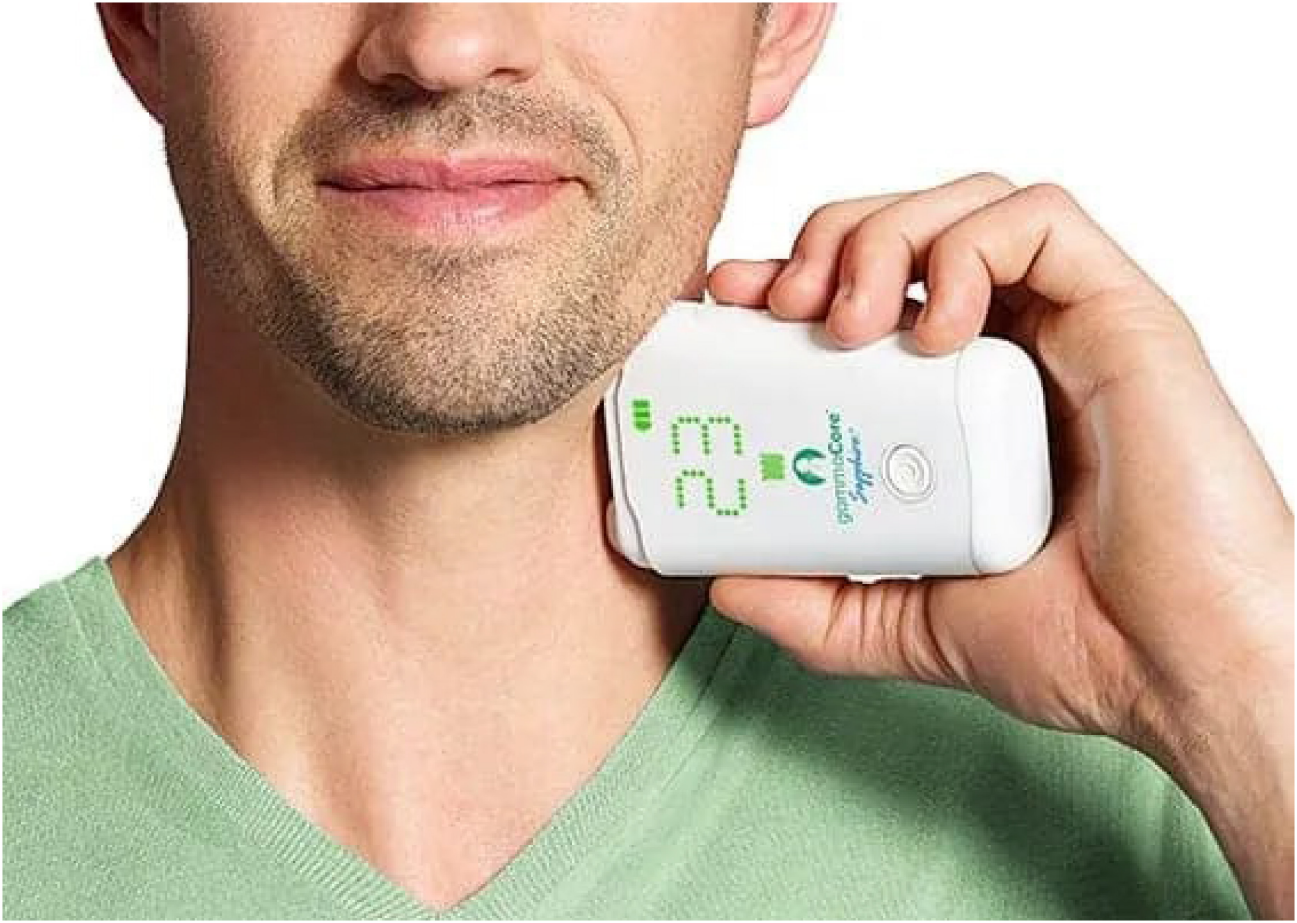
Application of transcutaneous cervical vagus nerve stimulation (tcVNS) using the gammaCore^®^ device. The bipolar electrodes are placed on the neck, aligned parallel to the underlying main trunk of the vagus nerve within the carotid sheath. This orientation is designed to maximize the activating function and facilitate the recruitment of consolidated Aβ-fiber bundles.

The most widely commercially available tcVNS device (gammaCore^®^, electroCore, Inc.), Figure 4, is a hand-held, rechargeable, portable device consisting of a rechargeable battery, signal-generating and -amplifying electronics, and a control button for the patient to control the signal amplitude. A pair of stainless-steel surfaces, which are the skin contact surfaces (stimulation surfaces), allow the delivery of a proprietary electrical signal. This signal consists of five 5000 Hz pulses repeated at a rate of 25 Hz for a maximum of 120 s per dose (U.S. Food and Drug Administration, 2017). The 5000 Hz frequency used is specifically designed to overcome skin impedance and penetrate the deep cervical fascia to reach the carotid sheath. While the carrier frequency is 5000 Hz, the burst frequency is 25 Hz, which aligns with the physiological firing rates used in taVNS and invasive VNS.

The waveform of the electric pulses approximates a sine wave with peak voltage limited to +/−30 Volts (24 Volts when against the skin of the neck) and a maximum output current of 60 mA. A conductive gel is applied to the stimulation surfaces to maintain an uninterrupted conductive path from the stimulation surfaces to the skin on the neck (electroCore Inc, 2020).

In the USA, gammaCore holds six distinct FDA-cleared indications in headache (electroCore Llc, 2018; U.S. Food and Drug Administration, 2018; U.S. Food and Drug Administration, 2017).

### TaVNS vs. tcVNS: anatomical targeting

The tragus of the ear is an interesting target for tVNS because the ABVN innervates the skin of the auricle and external acoustic canal (Kenny and Bordoni, 2025; Tekdemir et al., 1998). However, the ABVN is primarily a sensory nerve, making it hard to achieve consistent therapeutic results (Tekdemir et al., 1998). Several sensory nerves with variable fiber-density and significant overlap innervate the outer ear in addition to the ABVN: the auriculotemporal nerve, greater auricular nerve, and to some extent the lesser occipital nerve (Kadrie et al., 2025; Kaniusas et al., 2019) making auricular stimulation complex and triggering multiple reflexes, such as the ear-cough reflex (Arnold’s reflex) which may interfere with desired effects (Ryan et al., 2014). Consequently, the auricular branch consists of a diffuse network of superficial fibers with dispersed nerve endings that are difficult to target precisely, making taVNS inherently less focused (Tekdemir et al., 1998).

In contrast, the target of tcVNS is the main trunk of the vagus nerve, which is a large, well-defined, and consolidated bundle of nerve fibers located within the carotid sheath, typically positioned dorsomedially between the common carotid artery and the internal jugular vein (Hammer et al., 2018). This anatomical setup allows for uniform stimulation across the nerve bundle, because the vagus nerve runs directly under the treatment location (Garner et al., 2026).

- The large, direct access point provided by tcVNS may deliver a more consistent and powerful electrical signal to the central nervous system
- Clinical observations suggest this efficiency allows for significantly shorter stimulation times often as little as 2 min per session for tcVNS—whereas taVNS protocols frequently require 30–60 min of continuous or intermittent stimulation to achieve comparable neurophysiological changes (Jigo et al., 2024).

### taVNS vs. tcVNS: nerve fiber count

The vagus nerve is composed of fibers that can be classified by diameter into: A (Aα, Aβ, Aγ, Aδ), B, and C groups. Both taVNS and tcVNS require activation of Aβ-fibers, specifically, to elicit therapeutic effects (Patros et al., 2025; Yap et al., 2020a).

While A-fiber distributions within the carotid vagus have been well-documented, ABVN literature is sparse. Only one study to date has determined that the human ABVN contains thick myelinated axons (≥7 μm) belonging to the Aβ class, which are five to six times less numerous than those in the cervical nerve (Safi et al., 2016). Morphological studies show that the ABVN has just ∼1% of the number of myelinated fibers found at cervical level. Specifically, the CVN contains approximately 39,500 total nerve fibers [(35,100 myelinated + 4,400 unmyelinated), whereas the ABVN averages about 385 myelinated fibers (no unmyelinated fiber counts have been made to date)] (Hilz, 2022; Sachis et al., 1982; Tekdemir et al., 1998; Wang et al., 2023).

- The higher fiber count in the cervical region is believed to lead to a more robust and reliable response with tcVNS.

While some suggest that taVNS might preferentially activates the smaller, slower-conducting Aδ-fibers and C-fibers due to the different fiber composition of the auricular nerve and the lower current density of surface stimulation, leading to different therapeutic effects than tcVNS. But Stefan et al. (2012) showed that tVNS does not elicit painful sensations, indicating these fibers are not typically activated (Stefan et al., 2012).

### taVNS vs. tcVNS: electrode-axon orientation

Orientation of fibers relative to the stimulation electrode has a large effect on fiber recruitment. Fibers running parallel to the direction of the electrical current flow (and thus, the electrode) generally have lower activation thresholds and are more easily recruited than those oriented at an angle or perpendicularly to the stimulation contacts (Rattay, 1986, 1999). This is because the efficacy of electrical stimulation is determined by the change in the electrical potential —the activating function—along the nerve fiber. A parallel orientation maximizes this change in potential, facilitating depolarization. Optimizing electrode placement to align with the target fibers is a crucial parameter in maximizing the efficacy of electrical stimulation for therapeutic purposes, often having a greater influence on efficacy than other factors like electrode size (Frahm et al., 2016; Warman et al., 1992). The electrode contacts of tcVNS devices are oriented on the neck parallel to the direction of the target axons (Gurel et al., 2020). Meanwhile, there is no consistent or predictable orientation with respect to the individual axons and electrodes with taVNS due to the diffuse nature of the auricular nerve endings (Yap et al., 2020a). The difference in nerve structure and electrode orientation of tcVNS versus taVNS leads to fundamental differences in how stimulation affects the nerves.

- With cervical vagus nerve fibers oriented parallel to the direction of current flow, tcVNS creates the necessary voltage gradient more efficiently and leads to depolarization at lower stimulus thresholds.

### taVNS vs. tcVNS: comparative studies

Based on recent studies, ctVNS appears to be a more reliably effective method than aVNS.

A second-language acquisition study involving career linguists at the US Department of Defense’s Defense Language Institute found that tcVNS produced significant benefits in learning and mitigating fatigue, while taVNS did not show a notable effect in that specific paradigm (Miyatsu et al., 2024).

In a 2024 randomized, controlled, crossover pilot study, researchers directly compared the effects of cervical and auricular transcutaneous vagus nerve stimulation on the sensory performance of healthy adults. Results showed that tcVNS improved auditory performance by 37% visual performance by 23% compared to sham whereas, taVNS demonstrated no significant improvement. Furthermore, tcVNS showed clear physiological indicators of vagal engagement, such as changes in heart rate variability, which taVNS did not reliably produce (Jigo et al., 2024).

While preliminary head-to-head pilot studies suggest an advantage for tcVNS in specific sensory and cognitive paradigms, these findings are limited by small sample sizes. The primary distinction between the two modalities remains anatomical: tcVNS targets a consolidated, large-diameter nerve bundle with predictable electrode alignment, whereas taVNS engages a diffuse and variable terminal network. This anatomical consistency in tcVNS may lead to more reliable biophysical target engagement, though large-scale clinical trials are still required to establish definitive therapeutic superiority.

Beyond the studies on linguists and sensory performance, direct head-to-head comparisons remain relatively limited in the current literature. Animal studies directly comparing auricular and cervical stimulation revealed that each method activates distinct subregions and produces different spiking patterns in the Nucleus of the Solitary Tract (NTS; Yang et al., 2026). This provides cellular-level evidence that taVNS and tcVNS are not interchangeable and may lead to different therapeutic outcomes. It must also be noted that there do exist landmark positive trials for taVNS in epilepsy (Rong et al., 2016) and depression (Kong et al., 2018), showing its efficacy despite the anatomical complexity of the auricle and there are trials where tcVNS did not meet its primary endpoints, such as in Parkinson’s disease (Mondal et al., 2021).

### Side effects and discomfort

Transcutaneous cervical vagus nerve stimulation is generally well-tolerated, with most reported adverse events being mild and transient (Redgrave et al., 2018). In a large randomized controlled trial, the incidence of device-related application-site discomfort was approximately 2.5%, a rate comparable to sham stimulation (Tassorelli et al., 2018).

A significant percentage of participants in taVNS studies experience moderate levels of discomfort, including ear pain, skin irritation, and a tingling sensation. 16 The varied innervation of the ear by other nerves (trigeminal and greater auricular nerves) can lead to these localized, sometimes painful, sensations. The distracting or painful sensation from aVNS may interfere with the intended results by triggering non-specific somatic reflexes that confound vagally mediated outcomes (Yang et al., 2026).

## Discussion

Auricular and cervical vagus nerve stimulation have gained substantial interest across a wide range of clinical and wellness applications (Austelle et al., 2024; Hilz, 2022; Lv et al., 2022; Yap et al., 2020a). However, while both fall under the “transcutaneous” umbrella, they are fundamentally distinct in their anatomical targets, mechanisms of activation, and clinical profiles (Yang et al., 2026).

### Anatomical and biophysical distinctions

The primary distinction lies in fiber architecture and accessibility. The auricular branch of the vagus nerve (ABVN), or Arnold’s nerve, is superficial but contains a relatively small number of fibers (∼1% of the cervical count) and consists entirely of afferent projections (Bermejo et al., 2017; Butt et al., 2020; Kenny and Bordoni, 2025; Peuker and Filler, 2002; Safi et al., 2016). Consequently, taVNS relies on indirect, reflex-mediated pathways to influence central networks.

In contrast, tcVNS targets the main vagal trunk within the carotid sheath—a large, consolidated bundle containing a substantially higher population of myelinated Aβ fibers (Garner et al., 2026; Hammer et al., 2018; Patros et al., 2025; Sachis et al., 1982). This anatomical configuration facilitates more consistent biophysical target engagement (Gurel et al., 2020). Because the cervical fibers run parallel to the direction of current flow in tcVNS devices, they maximize the “activating function,” leading to depolarization at lower relative thresholds (Rattay, 1986, 1999; Warman et al., 1992). Conversely, the multidirectional and diffuse terminal network of the ABVN makes precise electrode–axon alignment unpredictable, which may contribute to the higher variability in clinical responses observed in auricular protocols (Frahm et al., 2016; Yap et al., 2020a).

### Technological and parameter variability

A primary challenge in isolating the efficacy of these modalities is the heterogeneity of stimulation parameters (Farmer et al., 2021). While taVNS typically utilizes constant-frequency stimulation (20–25 Hz), tcVNS employs a high-frequency carrier signal (e.g., 5000 Hz bursts) (electroCore Inc, 2020; tVNS Technologies GmbH, 2025). This high-frequency approach is a technical necessity to overcome skin impedance and penetrate deep cervical tissues to reach the carotid sheath (U.S. Food and Drug Administration, 2017, 2018). Furthermore, clinical claims by some devices regarding neck-based ABVN activation are anatomically implausible, as the ABVN does not descend into the cervical region (Butt et al., 2020; Kadrie et al., 2025). Such inconsistencies in terminology and parameter reporting complicate the interpretation of study results across the literature (Farmer et al., 2021; Yap et al., 2020a).

### Evaluation of comparative evidence

Preliminary head-to-head pilot studies have demonstrated measurable physiological effects with tcVNS—such as significant enhancements in sensory performance and language acquisition—that were not reliably observed with auricular approaches (Jigo et al., 2024; Miyatsu et al., 2024; Yang et al., 2026). However, these comparative studies are currently in the pilot stage and limited by small sample sizes (Yang et al., 2026). Furthermore, the clinical success of taVNS in domains like epilepsy and depression contrasts with neutral tcVNS findings in specific conditions like Parkinson’s (Kong et al., 2018; Mondal et al., 2021; Nemeroff et al., 2006; Penry and Dean, 1990; Rong et al., 2016; Stefan et al., 2012; Uthman et al., 2004; Yap et al., 2020b).

## Conclusion

Transcutaneous cervical vagus nerve stimulation offers a theoretical biophysical advantage due to its access to a consolidated nerve trunk and favorable fiber orientation, but both taVNS and tcVNS remain promising tools in neuromodulation. Future research must prioritize large-scale, high-quality clinical trials to define optimal parameters and identify the specific clinical contexts where the direct trunk engagement of tcVNS or the reflex-mediated pathways of taVNS are most effective.
